# Comparative study of innovative blends prepared by fortification of date powder to alleviate child malnutrition

**DOI:** 10.1002/fsn3.1862

**Published:** 2020-10-12

**Authors:** Nighat Raza, Muhammad Umair Arshad, Farhan Saeed, Umar Farooq, Ambreen Naz, Mian Shamas Murtaza, Huma Badar Ul Ain, Tabussam Tufail, Muhammad Imran, Faqir Muhammad Anjum

**Affiliations:** ^1^ Institute of Home & Food Sciences Government College University Faisalabad Pakistan; ^2^ Muhammad Nawaz Shareef University of Agriculture Multan Pakistan; ^3^ Faculty of Allied Health Sciences University of Lahore Lahore Pakistan; ^4^ University of the Gambia Gambia

**Keywords:** amino acid profiling, biological studies, date powder, PDCAAS, weaning food

## Abstract

Child malnutrition is one of the biggest problems in developing countries with higher level of food insecurity. Pakistan is 5th largest producer of date fruit; therefore, its processing and products should be explored in various dimensions. Being rich source of minerals and sugars, it can contribute in weaning foods in a good manner. In current study, three blends were prepared with specific proportions of spray‐dried date powder and rich in specific proportions. They were compared with each other and control (free of date powder). The nutritional profile of the formulations revealed that 100 g of each formulation included all macronutrients in compliance with the requirements of Food and Agriculture Organization for weaning formulations; moreover, they are enriched with minerals due to presence of date powder. The iron contents reached up to 12.74 ± 0.16 mg/100 g. The phosphorus, zinc, and potassium contents also increased with the increase in date powder subsequently. Physicochemical properties exhibited in compliance with the requirement of the weaning foods. The protein quality was assessed both in vivo and in vitro. Amino acid profiling indicated that the limiting amino acid in F1 and control were lysine but in F2 and F3 were threonine. This is suggested that date powder might contain good quality protein that was further explained in biological studies, the formulations that contained higher amount of date powder reveled better PDCAAS score 86.76 ± 4.5, true digestibility 84 ± 4.36, biological value 69.45 ± 0.69, net protein utilization 73.82 ± 1.46, and protein efficiency score 1.18 ± 0.07. The sensory evaluation revealed that F2 showed better result in overall acceptability. Thus, date powder is suggested to be used as good constituent that can fortify mineral contents and sugar contents of the weaning foods.

## Graphical abstract

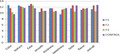

## 1 INTRODUCTION

The food use to complement breast milk either semisolid or solid from the age of six months is called as weaning food (Agostoni et al., 2008). Weaning foods are characterized as easily chewable and digestible foods with great energy density and possessing low bulk (Onweluzo & Nwabugwu, 2009). Traditionally, cereals are considered as most useful tools for fulfilling the energy and nutritional requirements of the children being caloric dense. But cereals are mainly deficient of lysine; an essential amino acid, but as they are full of other sulfur containing amino acids like methionine and cysteine, they can be used as complementary ingredient instead of a complete baby food itself (Ahmad *et al.,* 2014). Moreover, The commercially available weaning foods that are either imported or produced locally have quite higher cost due to sophisticated packaging, excessive processing, promotions, and marketing (Carvelho, Kenney, Carrington, & Hall, 2001; Fewtrell et al., 2017). The commercial weaning foods are not affordable for low‐income mothers, so homemade recipes are mostly preferable.

Plant‐based complementary formulations are mostly protein deficient, if they have adequate protein, the antinutrient contents in plants may not suffice available protein and they also suppress the absorption of many macro‐ and microminerals (Gibson, Perlas, & Hotz, 2006). The iron and calcium requirements should be fulfilled from complementary diets up to 50%–75%, likewise more than 25%–50% protein requirements of infants should be fulfilled from weaning food (Caulfield, Huffman, & Piwoz, 1999; Qasem, Fenton, & Friel, 2017). Therefore, it is the need of the hour in developing countries to produce a complementary food from locally available underutilized resources that is complete in all respects, for instance, it must be energy dense, having all nutrients according to RDA of growing children in order to fight against the problem of malnutrition among children of low socio‐economic groups (Sattar et al., 2013; White, Bégin, Kumapley, Murray, & Krasevec., J., 2017). Fruits are the source of immediate energy supply coupled with all sufficient amounts of minerals, vitamin, and handful of dietary fiber that is indispensable for a healthy gastrointestinal tract (Igah, 2008).

A blend of date powder and rice supplemented with whey proteins and whole milk powder could meet the requirements of current situation to cope the malnutrition issues in infants. The rice is documented to be easily digestible with low fiber, highly caloric density, and second most widely produced staple food in Pakistan therefore considered for the current study. Different blends were tried by researchers using brown rice, mung bean, and soybeans with yoghurt as dairy source and calculated the nutrient contents. Higher the contents of cereals and legumes, proteins and fat contents became higher (Munasinghe, Silva, Jayarathne, & Sarananda, 2013).

Therefore, as date powder contained three to four percent proteins and the extent to which utilized in the recipe was insufficient for infant requirements. Therefore, whey proteins being reliable and cheap protein source were used. Whole milk powder contained all those ingredients that were necessary for the growth and development of the growing infants. It was enriched with high‐quality proteins that could complement with the low protein and other nutrients enriched date flesh to produces a complete weaning food. Therefore, the aim of the study was the development of such nutritional blend for weanling kids that can fulfill their all requirements regarding proteins, essential amino acids, and micronutrients. Date powder blended with whey protein, whole milk powder, and rice flour could be the best source of food in their weanling age.

## 2 MATERIAL AND METHODS

### 2.1 Procurement of raw material

Whole milk powder was taken from Nestle, high‐quality brown rice from super market. Analytical grade chemicals were purchased from Merck, Oxide, and AppliChem.

### 2.2 Preparation of formulations

Rice flour slurry was prepared and cooked for 10 min, cooled, spread on a tray in the form of thin layer (1–2 mm) and oven‐dried (2 h at 90ºC). The flakes obtained were grounded in a blender. Spray‐dried date powder of aseel (Raza *et al.,* 2019), whole milk powder, whey protein concentrate, and all the ingredients were added and mixed well and finally sieved through 60mesh screen. The finished product was stored at 5ºC in polyethylene bags for further analysis.

### 2.3 Compositional analysis

Nutritional profile for ash, moisture, crude fat, protein, and NFE was done through respective methods (AOAC, 2000).

### 2.4 Mineral profiling of weaning foods

The procedure described in AOAC (1990) method No. 3.014‐016 was adopted to determine the mineral content. Na, K, was determined on flame photometer according to AOAC (2000). Whereas, Phosphorus contents was determined by spectrophotometry according to method given by Gul and Safdar (2009).

### 2.5 Caloric value

Gross energy value was measured by guidelines described in AOAC (2006) using Oxygen Bomb Calorimeter (C‐2000, IKA WERKE). Energy value of foods as a whole and per portion was determined. Passmore and Eastwood (1986) proposed guidelines that were followed for assessment of macromolecules energy by physiological fuel values implementing Atwater's conversion factors (4x% protein, 9x% fat). Additionally, nutrient density for respective proteins and fats was calculated per 100kcal (Food & Agriculture Organization/World Health Organization, 2006).

### 2.6 Physicochemical characteristics

The density was measured by adding specific amount of the weaning food in 100 ml volumetric flask and expressed as mass per unit volume. The sedimentation method was applied for determination of reconstitution index of complementary diets (Osundhunsi & Aworha, 2002). For reconstitution index, measured sample (1 g) was mixed with 10 ml distilled water and allowed to stand at ambient temperature (30 ± 2°C) for 30 min. The mixture was centrifuged for 30 min at 3,000 g. Water absorption was examined as percent water bound per gram weaning food (Sosulski, Garatt, & Slinkard, 1976). Rapid Visco Analyzer (Newport scientific, RVA surper 4, Australia) was used to assess the viscosity of 40% slurry of the weaning food. It was expressed as centipoise (Bukusuba, Muranga, & Nampala, 2008). In vitro starch digestibility and in vitro protein digestibility were determined according to respective methods (Ekwere, Igile, Ukoha, Mbakwe, & Anegu, 2017; Souilah et al., 2014).

### 2.7 Amino acid analysis

Weaning foods were analyzed for amino acid quantification through amino acid analyzer (model: Biochrom 30+, Physiological Systems, Biochrom Ltd.) according to method given by Maehre, Dalheim, Edvinsen, Elvevoll, and Jensen (2018).

### 2.8 Amino acid contribution

The one to two years infant's requirement for amino acids was calculated on an average basis (Reeds & Garlick, 2003; Food and Agriculture Organization/World Health Organization/United Nations University, 2007). The daily amino acid contribution (%) from weaning food was assessed according to the guidelines of Egounlety (2002).

### 2.9 Protein score

Essential amino acid score was recorded for complementary foods following the instructions of Food and Agriculture Organization/World Health Organization/United Nations University (2007) for preschoolers, the lowest score was expressed as protein score (Plahar & Hoyle, 1991).

### 2.10 PDCAAS value

Protein digestibility corrected amino acid score (PDCAAS) was calculated according to method of Kannan, Nielsen, and Mason (2001) by multiplying true digestibility of respective formulation and lowest value of amino acid score as per given in equation:

$$\text{PDCAAS}\;\left(\%\right)\;=\;\text{True digestibility}\;\times \;\text{lowest amino acid score}.$$

### 2.11 Housing of rats

For rats modeling, 40 weanling male rats were purchased from National Institute of Health (NIH), Islamabad, and kept in animal room, Department of Pharmacy, Government College University of Faisalabad. The rats were divided into four groups, 10 in each group. The rats were fed on formulation specified for a period of four weeks. The room temperature was maintained at 25ºC, and humidity was 50 ± 5% kept constant throughout the housing period with light and dark cycle of 12 hr. Urinary excreta, feces, and spilled diets were gathered throughout the study period, the urinary nitrogen and fecal nitrogen were calculated through kjeldahl apparatus. At the end of trial, rats were decapitated after keeping them overnight fast. The bodies of rats were weighed after removing of all organs and dried in oven for the calculation of Nitrogen assimilated in their bodies.

### 2.12 Formulation intake

Feed intake of each group was recorded by subtracting spilled plus offered diet from the total feed consumed on daily basis during the study period (Wolf & Weisbrode, 2003).

### 2.13 Body weight gain

Total gain in body weight of individuals was calculated. Then mean of each group was taken to measure the growth index for specified formulations, the gain in body was calculated with interval of 8, 16, 21, and at the termination of the study period (Seena, Sridhar, Arunb, & Young, 2006).

### 2.14 Protein quality evaluation

The quality of protein was assessed according to method established by Ingbian and adegoke (2007) by calculating the growth study parameters:

- Protein Efficiency Ratio
- Net Protein Ratio
- Relative Protein Ratio
- Nitrogen Balance Study

Nitrogen balance study was done after getting the nitrogen contents from spilled diets, urinary excreta, feces, and dried rat bodies. The calculation of true digestibility (TD), biological value (BV), and net protein utilization (NPU) was done using nitrogen contents.

### 2.15 Safety evaluation

#### 2.15.1 Blood sampling

After overnight fasting, the rats were anesthetized and blood collected by jugular vein cannulation (Parasuraman, Raveendran, & Kesavan, 2010) into EDTA‐coated sterile containers. For ALP test, the container must not be coated with EDTA.

#### 2.15.2 Liver and kidney functioning test

Serum alanine aminotransferase (ALT), alkaline phosphatase (ALP), and aspartate amino transferase (AST) enzymes are the marker of liver function tests and were performed to evaluate current condition of the liver. Similarly, urea and creatinine quantitation are the markers of kindey functions and were performed in semi‐automatic analyzer (Mini techno, USA) using individual kits for each assay.

#### 2.15.3 Sensory evaluation

All the formulations were evaluated for various sensory characteristics by trained taste panel following 15 cm unstructured line for apparent and taste characteristics (Meilgaard, Civille, & Carr, 2007). The format for sensory evaluation was comprised of three parts; dry mix evaluation for visible characteristics; apparent characteristics of reconstituted formulations for color, texture, aroma, ease of preparations, thickness, and smoothness while third portions constitute evaluation of taste attributes like sweetness, flavor, taste, and overall acceptability. Periodic evaluation was done in Food analysis lab, Institute of Home and Food Sciences, GCUF. The formulations were offered to the panelist in transparent cups, water for reconstitution in cups was also provided while water to clear the taste between the sample evaluations was also provided with plain water. The samples were provided randomly to rate their acceptance by marking a cross on the line for all sensory traits. The obtained data by using metric scale were converted to numerical scores.

### 2.16 Statistical analysis

The obtained data of each parameter were subjected to factorial design using Statistical Package (Statistix 8.1.) Levels of significance were determined through ANOVA using CRD and means were compared through LSD.

## 3 RESULT AND DISCUSSION

### 3.1 Proximate composition

All three formulations showed highly significant difference among the protein levels (Table 1). The quantity of proteins in control weaning food was 10.6 ± 0.3 g/100 g while F1 exhibited a better quantity of 14.9 ± 0.7 with subsequent decrease in the rice quantity and increase in date powder in F2 and F3 the quantity of proteins became lower and showed the values of 12.6 ± 0.4 and 10.6 ± 0.4 g/100 g. The crude fat contents of formulations varied significantly, the lowest amount was assessed in control 8.95 ± 0.25 while F1, F2, and F3 with values 14.4 ± 0.5%, 13.3 ± 0.5%, and 12.8 ± 0.3% respectively. The crude fiber contents in control were 2.52 ± 0.06%, while it showed an increasing trend in the formulations as F1 contained 3.4 ± 0.1% and the F2 and F3 3.9 ± 0.3% and 4.2 ± 0.0, respectively. This increase was due to higher concentrations of date powder in F2 and F3 as compared to F1. The ash content increases significantly among the formulations. As the ash percentage in control was 2.86 ± 0.05 and F1, F2, and F3 were 3.7 ± 0.0%, 4.2 ± 0.0%, and 5.3 ± 0.0%, respectively. The value of NFE represents all digestible carbohydrates. They also revealed an increasing trend as the date powder increases even 10%. In control NFE was assessed as 62.4 ± 0.46% and in F1, F2, and F3 were 65.1 ± 0.2%, 66.2 ± 0.4% and 67.1 ± 0.2%, respectively. All these formulations despite of variation among each other are still in agreement with the Food and Agriculture Organization/World Health Organization (2006) standards for complementary diets. According to Food and Agriculture Organization/World Health Organization (2006), the recommended daily allowance of the 7 months and older infants was 11 g/day and a complementary food should contain at least 12–15 of the total proteins where fat contents of complementary foods should be in the range of 10%–25%. The RDA of fat contents for infants older than 7 months is 30 g/day that can meet the nutritional requirements of the growing bodies (Achidi et al., 2016).

**Table 1:** Formulations for weaning foods

Ingredients	Control	F1	F2	F3
Rice Flour _(g)_	70	40	30	20
DP (g)	0	30	40	50
WMP _(g)_	15	15	15	15
WPC (g)	5	5	5	5
Corn Oil(ml)	5	5	5	5
Nuts powder (g)	5	5	5	5
Ronoxen A	0.01	0.01	0.01	0.01
NaFe EDTA	0.0	0.1	0.1	0.1
Total	100	100	100	100

Abbreviations: DP, Date powder; WMP, Whole milk power; WPC, Whey protein concentrate.

### 3.2 Minerals

The values (Table 2) revealed the range of calcium varied between 324.75 ± 0.13 for F2 to 3.27.85 ± 3.37 and the control formulation contained 189.19 ± 0.84 mg/100 g of calcium. But iron content in the controlled formulation was 4.37 ± 0.08 mg/100 g while in investigative formulation it varied between 12.40 ± 0.40 for F1 and F2 while F3 formulation disclosed comparatively higher, that is, 12.74 ± 0.16 mg/100 g. Such higher contents in the formulations were suggestive of the Na Fe EDTA addition as fortificant. Phosphorus contents were not significantly different and ranged between 447.65 ± 0.91 and 452.45 ± 0.52. The zinc contents of formulations were higher than the control because the date powder and rice powder both were good source of zinc minerals, and the higher content in the infant formulations gave very positive results regarding the intestinal health of the infants, because it is established fact that the elemental zinc can make a coating on the intestinal mucosal line and help it to make a barrier for pathogen attack (Bajait & Thawani, 2011). According to Codex Alimentarious 3.2 mg/100 g is per requirement for zinc whereas our formulation can provide up to 9.83 ± 0.04 mg/100 g of the zinc, there is no evidence that higher quantities of zinc if administrated can harm in any it was way (Larson, Roy, Khan, Rahman, & Qadri, 2008). The potassium contents were higher in the date powder; therefore, it contributed in the formulation as well, highest contents were found in F3 formulation with value of 1,201.96 ± 0.89 of K which is followed by F2 with value 1,195.90 ± 0.23 mg/100 g and F1; 1,187.69 ± 0.74 mg/100 g. The mineral contents in the control were lesser except phosphorus where it was almost equal to those of formulations and in case of sodium it was highest with value 144.44 mg/100 g. Zinc, magnesium, and manganese contents were lowest as per the findings of the study.

**Table 2:** Nutritional and physicochemical profiling of formulations and control

Indices	Control	F1	F2	F3
PROTEIN (%)	10.6 ± 0.3	14.9 ± 0.7	12.6 ± 0.4	10.6 ± 0.4
FAT (%)	8.95 ± 0.25	14.4 ± 0.5	13.3 ± 0.5	12.8 ± 0.3
ASH (%)	2.86 ± 0.05	3.7 ± 0.01	4.2 ± 0.03	5.3 ± 0.01
FIBER (%)	2.52 ± 0.06	3.4 ± 0.1	3.9 ± 0.3	4.2 ± 0.0
NFE (%)	70.4 ± 0.46	65.1 ± 0.2	66.2 ± 0.4	66.2 ± 0.4
ENERGY VALUE	411.93 ± 1.60	402.22 ± 0.88	409.48 ± 0.17	415.97 ± 0.33
Proteins (g/Kcal)	2.04 ± 0.07	3.71 ± 0.16	3.07 ± 0.09	2.56 ± 0.09
Fat (g/Kcal)	2.07 ± 0.16	3.58 ± 0.12	3.24 ± 0.12	3.24 ± 0.12
Ca (mg/100 g)	189.19 ± 0.84	222.74 ± 0.16	324.75 ± 0.13	325.61 ± 0.41
Fe (mg/100 g)	4.37 ± 0.08	12.40 ± 0.14	12.40 ± 0.14	12.74 ± 0.16
P (mg/100 g)	447.65 ± 0.91	452.45 ± 0.52	448.99 ± 0.68	450.82 ± 0.15
Zn (mg/100 g)	2.84 ± 0.06	9.83 ± 0.04	9.81 ± 0.03	9.45 ± 0.09
K (mg/100 g)	865.00 ± 1.35	1,187.69 ± 0.74	1,195.90 ± 0.23	1,201.96 ± 0.89
Na (mg/100 g)	140.44 ± 0.39	98.45 ± 0.29	98.27 ± 0.06	98.45 ± 0.09
Mg (mg/100 g)	48.85 ± 0.11	80.58 ± 0.23	81.40 ± 0.17	82.31 ± 0.16
Mn (mg/100 g)	2.06 ± 0.08	10.75 ± 0.17	10.71 ± 0.32	10.70 ± 0.15
Reconstitution Index (ml)	53.17 ± 0.31	52.1 ± 0.3	53.7 ± 0.6	53.2 ± 0.3
Water holding capacity (ml/g)	0.88 ± 0.05	0.71 ± 0.03	0.85 ± 0.03	0.94 ± 0.05
Viscosity (cP)	2093.4 ± 7.14	2,105.3 ± 7.1	2,113.8 ± 1.6	2,117.9 ± 0.4
Bulk Density (g/cm^3^)	0.57 ± 0.04	0.48 ± 0.03	0.61 ± 0.02	0.61 ± 0.02
IVPD	82.3 ± 0.1	87.2 ± 0.3	86.8 ± 0.5	86.8 ± 0.5
IVSD	72.63 ± 0.45	77.6 ± 0.5	77.8 ± 0.4	74.2 ± 0.4

Note

Values are mean ± standard deviation; results are means of three replicates.

### 3.3 Physicochemical properties of weaning foods

#### 3.3.1 Reconstitution index

Table 3 depicted only variation in F1 formulation compared to the other formulations and control. The results revealed that all three formulations showed 52.1 ± 0.3 ml of water required for consistent reconstitution. The mean values for F2 and F3 were 53.7 ± 0.6 and 53.2 ± 0.3 respectively while result for control was in close agreement with that of F2 and F3 with mean value of 53.17 ± 0.31 ml. The reconstitution indices of processed and unprocessed weaning foods were determined by researcher and found that RI of unfermented weaning food was always lower than fermented weaning foods ranged from 65ml to 86ml, while in unfermented weaning foods ranged varied from 45ml to 54ml (Onilude, Sanni, & Ighalo, 1999).

**Table 3:** Biological evaluation for protein quality estimation

Biological test	Control	F1	F2	F3
PDCAAS	64.28 ± 0.89	86.76 ± 4.5	77.04 ± 0.98	76.90 ± 0.56
True digestibilty	84.1 ± 1.15	84 ± 4.36	79 ± 1	80 ± 1.1
Biological value	78.31 ± 0.35	69.45 ± 0.69	69.22 ± 0.94	69.18 ± 0.75
Net protein utilization	82.29 ± 0.85	73.82 ± 1.46	78.79 ± 0.56	64.46 ± 0.72
Protein efficiency ratio	2.27 ± 0.17	1.18 ± 0.07	2.25 ± 0.12	2.59 ± 0.31
Net protein ratio	3.38 ± 0.04	1.87 ± 0.12	3.15 ± 0.03	3.60 ± 0.21
Reference NPR	84.78 ± 0.46	58.93 ± 1.61	61.71 ± 1.38	70.86 ± 0.61

#### 3.3.2 Water holding capacity (WHC)

The means ± *SD* showed that control and F2 formulations are having nearly equal water holding capacities with values 0.88 ± 0.05 ml/g and 0.85 ± 0.03 ml/g, respectively. While with the higher contents of date powder, the WHC in F3 were increased to 0.94 ± 0.04 ml/g and the lowest were reported in F1 formulation with value 0.71 ± 0.03 ml/g.

According to Usman, Bolade, and James (2016) found much higher levels of WHC of weaning foods, and the reason for higher WHC was only cereal and soybean‐based formulations, higher the protein contents in some gruels, higher will be the WHC. In a similar study, WHC of weaning food from sorghum was assessed as 0.44 ± 0.14 ml/g (Lalude & Fashkin., 2006).

#### 3.3.3 Viscosity

Higher viscosities are associated with larger gruels, difficulty swallowing, and too much particles binding with each other. Mean values of viscosities of different blends showed highly significant difference among each other. The viscosity of the control was 2093.4 ± 7.14cP, while it amplified in the formulations with increase in the concentration of date powder and reached to 2,117.0 ± 0.4 in F3. Mostly viscosity of complementary food for infants required between 1,000 and 3000cP. Higher viscosities could hamper the absorption and digestion of the nutrients, whereas very lower viscosity could make trouble with the spoon feeding to the infants and decrease in nutrient bulk, in particular, quantity of feed. In many cereals and legumes like pigeon pea and millet, their fermented forms and blends were compared with the commercial diets but their viscosity was much lower than that of the commercial food, that is, it was ranged from 200 cP to 220cP while in control it was 3030cP (Onweluzo & Nwabugwu, 2009).

#### 3.3.4 Bulk density

The mean values of all formulations significantly differ in F2 and F3 with values 0.61 ± 0.02 g/cm, while minimum was in F1. This decrease was suggestive of less quantity of date powder. Different blends of soybean flour and cocoyam starch were analyzed for loose and packed bulk densities. All the blends showed same packed bulk densities whereas loose bulk density was varied, the blend that contained 20:80 ratio of soybean and modified cocoyam starch showed highest loose bulk density with value of 0.5 ± 0.0 whereas all other blends showed equal loose bulk densities with values of 0.34 g/cm (Ojinnaka, Ebinyasi, Ihemeje, & Okorie, 2013).

Lalude and Fashakin (2006) prepared different experimental diets and compared with the Nutrend (commercial diet) and found no less bulk density, as experimental diets contained mean value of 0.657 ± 0.02 g/ml and Nutrend contained 0.605 ± 0.16 g/ml.

#### 3.3.5 Nutrient density

The quantification of the major nutrients and their expression on calorie basis with respect to the total energy uptake from the weaning food can be helpful to determine whether the particular weaning food can provide the individual nutrients according to standards/as per physiological requirements (Mosha & Vicent, 2004). The results found significant difference among the protein contents provided by the three formulations and control, whereas the fat contents were higher is subsequent formulations compared to control that has value of 2.74 ± 0.16 g/100 kCal; protein contents of control were 3.54 ± 0.07. Among all formulations, the highest fat and protein content was provided by F1 with quantity of 3.58 ± 0.12 g/100 Kcal and 3.71 ± 0.16 g/Kcal of fat and proteins respectively.

According to Food and Agriculture Organization/World Health Organization (2006), the energy provision in a weaning food from protein and fat must not exceed to 5.5 g/100 Kcal and 4.5 g/100 kcal, respectively. The findings in the current study are in parallel with these guidelines.

#### 3.3.6 In vitro*protein digestibility*

The significant variations were observed among the protein digestibility values obtained from control and that of formulations. Among all three formulations, little difference was observed. The value for F3 was 86.2 ± 0.3 while for F1 and F2 were 87.2 ± 0.3 and 86.8 ± 0.5, respectively. These results are suggestive of the good quality protein being provided by the prepared weaning foods because amino acid assimilation was quite good. The higher value of IVPD is due to good processing of the formulations and the presence of digestible proteins like whey proteins and precooking of brown rice showed similar to the finding of different scientists (Jood, Chauhan, & Kapoor, 1988; Kataria, Chauhan, & Punia, 1989).

#### 3.3.7 In vitro*starch digestibility*

In current study, brown rice was precooked before making slurry for drying therefore the findings are according to the formulations showed good IVSD values. All three formulations did not show significant difference. Comparatively lower value was observed in control.

#### 3.3.8 Amino acid profile

The quality of protein is mostly assessed by the number and quantity of essential amino acid present in the protein. The highest contents of ARA (82.73 ± 0.34) compared to F2 (81.74 ± 0.41) and F1 (80.07 ± 0.59) were estimated but the control contained minimum among all four diets with value 59.27 ± 0.47. Similar pattern was followed by sulfur containing amino acids (methionine + cysteins) and were found highest (30.76 ± 0.43) in F1 and minimum 15.24 ± 0.08 in control sample. As the concentration of date powder increases in the formulations the lysine contents increase and reached up to 68.24 ± 0.5 in F3 formulation followed by F2 and F1 and at the end minimum contents were found in control with value 23.21 ± 0.36. Isoleucine contents also lesser in control diet as showed in the related table of means + *SD*. The best isoleucine contents were found in F2 formulation with value 48.27 ± 0.58, and histidine was found highest in control (27.66 ± 0.33) followed by F1 (25.76 ± 0.36) and F2 (23.15 ± 0.39). The tryptophan contents were lowest in F1 (15.33 ± 0.73) and highest in F3 (40.04 ± 0.61) were suggestive of the less tryptophan contents in rice protein whereas date protein is excellent source of tryptophan. The valine contents were highest in F1 but compared to control all formulations were having lesser contents. The total amount of essential amino acids were highest in the F1 formulation followed by F3 (411.11 ± 2.40) and F1 (410.43 ± 2.02). The lowest were reported in control. So we could claim that the prepared weaning foods were having better amino acid profile than the control. The weaning food along with essential amino acid also provides the nonessential amino acids, the arginine contents of F3 were highest reported with value 84.36 ± 0.63, and the other two formulations were also not differing too much but the lowest amount were reported in control. The alanine, aspartic acid, glutamate, proline, and serine followed the same pattern or increasing order in the formulations with the lowest in F1, that is, 58.48 ± 0.52, 122.33 ± 0.85, 141.72 ± 0.44, 31 ± 0.39, and 25.84 ± o.5, respectively. The alanine contents were lowest in the control whereas glutamate was highest in F3 and in control its quantity was 169.58 ± 0.58. The most abundant nonessential amino acids were found in F3 formulations with value 618.56 ± 0.61 followed by F2 (562.85 ± 0.41) and F1 501.65 ± 0.45. The amount of total nonessential amino acids in control was up to 540 mg/100 g.

The quantity of essential amino acids was very much crucial in proteins for better quality evaluation. The essential amino acid being lack of their precursors in the body is indispensable to be provided by external source for better growth and development particularly in infancy where development of better immune system is equally important (Adeyeye & Afolabi, 2004; Wu, 2010). Lysine is mostly the limiting amino acid in most of the cereal‐based weaning foods, similarly maize is deficient source of tryptophan and other sulfur containing amino acids, so the combinations of legumes and cereals were used that proved good source of such limiting amino acids (Ijarotimi & Olopade, 2009; Mune, Minka, Mbome, & Etoa, 2011.

#### 3.3.9 Amino acid score

The findings showed better amino acid scores by F1 and F2 formulations compared to F3, the amino acid score of the control was also almost equivalent to that of F1 and F2. These finding revealed that a good combination of date powder with rice flour could generate a quality weaning food that provide good quantity of essential amino acids. The F1 and control were showing lysine as limiting amino acid which could predict the higher concentration of rice flour which is deficient of lysine. Threonine was found as limiting amino acid in F2 and F3 formulations.

**FIGURE 1:**
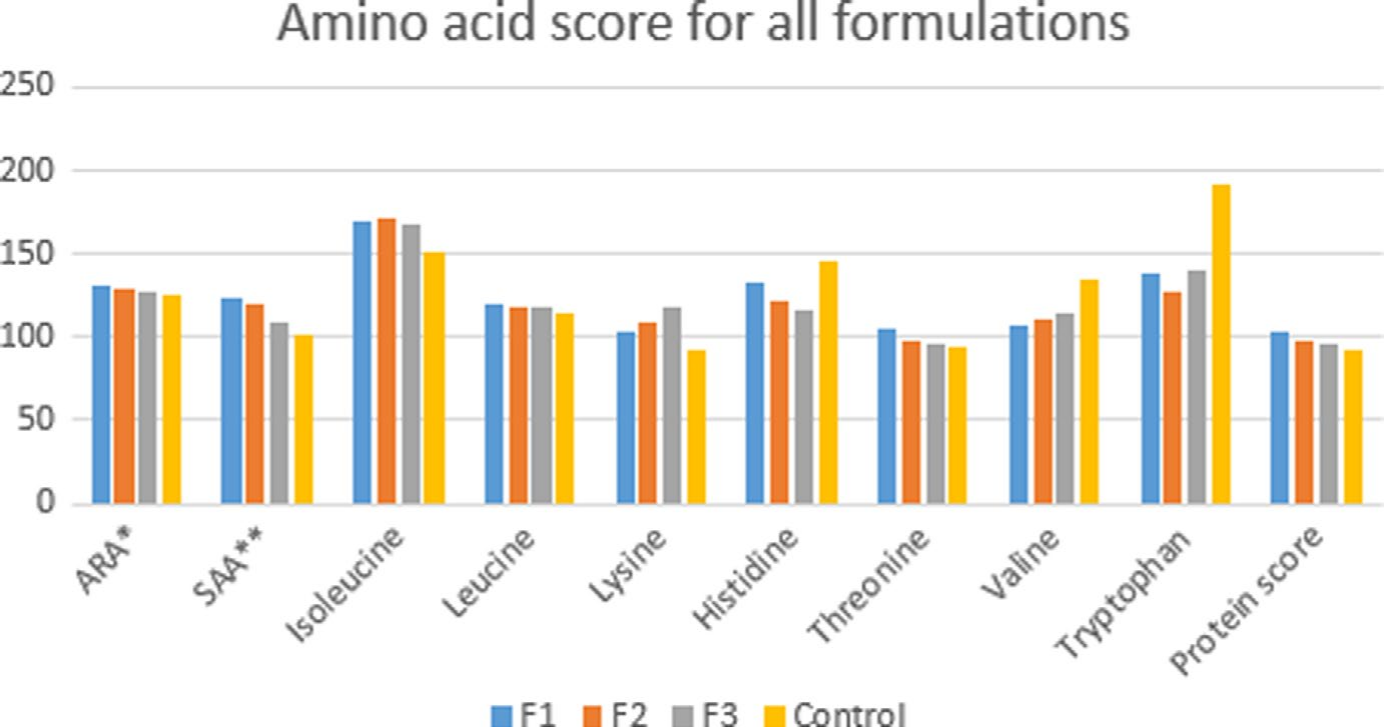
Amino Acid score for all formulations. *Aromatic Amino acid (Phenylalanine + Tyrosine). **Sulfur containing amino acids (Methionine + Cysteine). ***LAA (Limiting Amino acids)

Similar results were found by many scientists who used cereal as staple food for the preparation of weaning foods (Juliano & Hicks, 1996). It is evident from literature review that processing techniques like cooking and soaking could also affect the availability of lysine contents (Seena et al., 2006). Moreover, the second limiting amino acid was threonine in F1 and control and in F2 and F3 s limiting amino acid was lysine. So, all three formulations contain lysine and threonine in lesser quantities compared to the other amino acids (Table 4, Figure 1).

**Table 4:** Amino Acid Concentrations in all formulations

Amino Acid	Control	F1	F2	F3
ARA	59.27 ± 0.47	82.73 ± 0.34	81.74 ± 0.41	80.07 ± 0.59
SAA	15.24 ± 0.08	30.76 ± 0.43	29.91 ± 0.30	27.16 ± 0.56
Isoleucine	32.45 ± 0.40	47.44 ± 0.66	48.27 ± 0.58	46.91 ± 0.29
Leucine	46.09 ± 0.43	79.49 ± 0.57	78.12 ± 0.69	78.15 ± 0.38
Lysine	23.21 ± 0.36	59.90 ± 0.30	63.12 ± 0.45	68.24 ± 0.50
Histidine	27.66 ± 0.33	25.76 ± 0.36	23.15 ± 0.39	22.25 ± 0.78
Threonine	28.06 ± 0.45	36.05 ± 0.20	14.05 ± 0.36	15.47 ± 0.83
Valine	27.44 ± 0.66	37.30 ± 0.85	33.10 ± 0.25	32.82 ± 0.64
Tryptophan	22.06 ± 0.33	15.33 ± 0.73	38.97 ± 0.45	40.04 ± 0.61
TEAA	281.48 ± 1.86	414.77 ± 1.86	410.43 ± 2.02	411.11 ± 2.4
Arginine	81.89 ± 0.34	82.82 ± 0.42	83.47 ± 0.65	84.36 ± 0.63
Alanine	57.18 ± 0.72	58.48 ± 0.52	65.89 ± 0.62	72.90 ± 0.54
Aspartate	131.91 ± 0.31	122.33 ± 0.85	129.03 ± 0.68	134.66 ± 0.67
Glutamate	121.58 ± 0.58	141.72 ± 0.44	180.5 ± 0.63	210.84 ± 0.5
Glycine	27.03 ± 0.45	40.82 ± 0.43	41.68 ± 0.49	40.86 ± 0.48
Proline	17.51 ± 0.37	31 ± 0.39	34.61 ± 0.63	39.78 ± 0.46
Serine	14.60 ± 0.63	25.84 ± 0.5	30.63 ± 0.52	36.72 ± 0.51
TNE	540.55 ± 0.67	501.65 ± 0.45	562.85 ± 0.41	618.56 ± 0.61

Note

Values are mean ± standard deviation; results are means of three replicates.

#### 3.3.10 PDCAAS value

The concept of protein deficiency corrected amino acid score was first time adopted in 1993 in order to evaluate the protein quality by comparing it with another reference protein on the basis of delivery of indispensable amino acids in the human body, and simultaneous determination of true digestibility of the test protein and its ability to fulfill the requirement of essential amino acids.

It is obvious from the table value for PDCAAS that all three formulations differ for their amino acid profile and their assimilations as well. The maximum PDCAAS value was exhibited by F1 with value 86.76 ± 4.5 and lowest by the control. The F2 and F1 were showing the similar PDCAAS value suggesting almost equal digestibility and amino acid profile.

Good digestibility of protein and maximum assimilation of requisite amino acids resulted into better PDCAAS value, whereas comparatively lesser values as observed for F2 and F3 formulations evident of poor absorption and lesser provision of indispensable amino acids from those formulations. These formulations follow the criteria of minimum 70% PDCAAS score for complementary diets (Muoki, de Kock, & Emmambux, 2012). In another study, the commercial and locally prepared complementary foods were administrated to Sprague Dawley weanling rats for nutritional and biological assessment revealed almost equal PDCAAS score that proposed about the effectiveness of local produce for the production of weaning foods because they are not less than the industrially prepared commercial diets (Amagloh et al., 2015; Mosha & Vicent, 2004).

### 3.4 Biological evaluation of weaning foods

The nutritional assessment was observed through proximate composition and amino acid profiling of the test formulations and comparison in the above section was quite evident. The biological study gave us more deep insight of those in vitro indices. The biological study was subdivided into three sections where Nitrogen balance study was carried out by calculating true digestibility (TD %), biological value (BV %), and net protein utilization (NPU %). Nutrient response growth study was carried out by calculating PER (protein efficiency ratio), NPR (net protein ratio), and RNPR (relative net protein ratio).

Mean squares for nitrogen balance included true digestibility (TD%), biological value (BV), and net protein utilization (NPU) study showed highly significant variations among all three formulations and control as well as minimum value of TD in F2 formulation was observed with value 79 ± 1, whereas the F1 and F3 generated 84 ± 4.36%, and 80 ± 1.1% respectively. The TD of control was equal to the F1. But according to Food and Agriculture Organization guidelines (2012) minimum TD for complementary foods should not be <70%. Despite of internal difference among all formulations, they were not below the standard to Codex Alimentations. It is established fact from earlier studies that better digestibility results in better assimilation and reduce the fecal nitrogen content by maximum retention of nitrogen in the body. The true digestibility can be enhanced by the addition of limiting amino acid in the test protein (Kamau, Serrem, & Wamunga, 2017; Mepba & Achinewhu, 2003). The biological value of test proteins means the ability of absorbed proportion of protein from the food to become part of the body for muscular development. The minimum conversion of protein nitrogen to fecal and urinary nitrogen resulted into better BV value. So higher the BV of proteins, greater will be the ability to become part of the body. It was evident from the result that all three formulations were having almost equal BV but the control value was quite higher suggesting good nitrogen retention from the absorbed protein in the food. There could be many factors that can affect the digestibility and bioavailability of the provided proteins. Many processing techniques like heat treatments improve the availability and proteins; moreover, the presence of good quality proteins containing all indispensable amino acid and physiological deficiency of those amino acids can also enhance the BV of the given protein. The study of African yam beans revealed their biological value was equal to 59.5% (Onwuka, Catherine, Jude, & Ayalogu, 2010). The biological value or the rates at which protein deposited in the body have direct relationship between dietary intake of proteins and nitrogen retention for the subjected sources of proteins that further strengthen the importance of biological value. This is then used to calculate the average dietary protein requirement (Reeds & Garlick, 2003). The results suggested highly significant difference for all the formulations with maximum NPU was from control diet (82.29 ± 0.85%) followed by F2 (78.79 ± 0.56) and F1 (73.82 ± 1.46). The minimum NPU was evident from F3 with value 64.46 ± 0.72. It is obvious from the results that net utilization of absorbed protein and nitrogen retention in Sprague Dawley rats was best in control and when it came to formulations the maximum absorbance of nitrogen from digested food was from F2 formulation. But the proteins from F3 could not be retained to that extent suggesting that the date powder was not a very good source of proteins because the highest contribution of date powder was in F3 formulation. Conclusively, it can be assumed from nitrogen balance indices that all the diets made from date powder and processed brown rice powder were good source of dietary proteins with maximum amount of indispensable amino acids, although values fluctuated to some extent but it was worthless because the cutoff values lied in the standard value.

### 3.5 Growth study parameters

Significant variations were observed among all formulations for providing sufficient proteins required for the growth and body weight gain of test animals. The major role of consumed proteins is growth and maintenance, if sufficient energy bulk is provided from carbohydrates sources. In the absence of sufficient energy source, proteins provide the required energy. The PER value is indication of quality of protein that whether protein is digested and assimilated properly and whether it added weight to the test animal. The assessment of growth study parameter in animals was suggestive of same results in humans. To incorporate in some complementary diets for infant PER should not less than 70 percent of the reference pattern provided by Food and Agriculture Organization/World Health Organization (2006). In current study lowest PER was showed by control. Highest PER was showed by F3 formulation with value (2.59 ± 0.31) followed by control (2.27 ± 0.17) and F2 (2.25 ± 0.12). The minimum value of PER was deducted from F1 (1.18 ± 0.07). The highest value from F3 means the rats fed on that diet performed well in terms of body weight gain. But overall all formulations value ranged above 2.25 PER value were suggestive of their good amino acid profile and utilization by Sprague Dawley rats at their best because reference protein casein has PER equivalent to 2.7 (Food & Agriculture Organization/World Health Organization, 2006). The PER and RNPR value of many animal‐based weaning formulations were also showed improved results because of the absence of antinutritional factors unlike plant sources where antinutritional sources always need to combat first (Hoffman & Falvo, 2004). The animal sources are always better with their PER value like beef, egg, and whey protein have 2.9, 3.3, and 3.2 PER, respectively. Unlike PER net protein utilization is the direct measurement of the net utilization of the protein by the test animals. There is significant difference of NPR among all the formulations and control. Highest NPR was evident from control showing maximum utilization of the proteins from commercial weaning food. Among prepared formulations, highest NPR value was from F3 formulation with value 3.60 ± 0.21 whereas F2 and F1 gave lesser ratio with value 3.15 ± 0.03 and 1.87 ± 0.12 respectively. Conclusively, maximum protein contents utilization by F3 suggested the excellent quality of date powder protein. The RNPR is a similar parameter to NPR but results were exhibited as ratio between the NPR values of test proteins to the standard reference proteins, that is, casein which is considered as 100. Like NPR the highest value of relative net protein utilization was deduced from control (84.78 ± 0.46) followed by F3 with value 70.86 ± 0.61. F2 and F1 revealed 61.71 ± 1.38 and 58.93 ± 1.61 values, respectively.

### 3.6 Kidney functioning tests

The urea and creatinine determination are the key indicators for monitoring the renal functioning. All four groups that were fed on formulations were tested for these indicators. There was very little fluctuation in the results. The weanling animals usually required higher nitrogen contents and maximum assimilation of nitrogen from the proteins for body buildups. And urea contents in blood are due to deamination of proteins. Consequently, less quantity of urea was noticed in the samples. The values for urea were ranged between 11.95 ± 0.63 to 13.30 ± 0.8 mg/dl, a similar trend was observed in creatinine where values were ranged between 0.32 ± 0.2 to 0.33 ± 0.02 mg/dl. Mean squares in Table 5 also showed nonsignificant difference among the groups fed on formulations and control as well.

These results are in the normal range of urea and creatinine, so it is evident from the results that formulations had no adverse effect on the rats therefore, safe to consume (Table 5).

**Table 5:** Mean ± *SD* of kidney and liver function test in Sprague Dawley rats

Formulations	Control	F1	F2	F3
Urea (mg/dl)	11.95 ± 0.73	13.30 ± 0.80	12.33 ± 0.65	12.54 ± 0.63
Creatinine (mg/dl)	0.32 ± 0.02	0.33 ± 0.02	0.33 ± 0.02	0.35 ± 0.02
ALT(IU/L)	38.38 ± 0.70	40.44 ± 2.44	41.34 ± 0.85	40.99 ± 1.11
AST (U/L)	77.20 ± 0.75	82.79 ± 0.47	82.73 ± 0.58	82.02 ± 0.94
ALP (U/L)	136.53 ± 1.90	147.30 ± 3.60	145.15 ± 1.76	145.74 ± 1.87

### 3.7 Liver functioning tests

The serum levels of alanine phosphatase (ALP), aspartate transaminase (AST), and alanine transaminase (ALT) were the indicator of hepatic functioning. The tests performed for serum of Sprague Dawley rats fed on formulations and control at the termination of the trial revealed no significant difference among all three formulations and control as well. There is minor fluctuation of ALP among the three formulations and value ranged between 40.44 ± 2.44 and 41.34 ± 0.85 IU/L, and the control showed little lesser value 38.30 ± 0.7 IU/L. Similar trend was exhibited by the AST with values 82.02 ± 0.94 to 82.79 ± 0.47 U/L the lowest value was reported for control, that is, 77.20 ± 0.75 U/L. Serum ALP was fluctuated between 136 ± 1.90 to 147.30 ± 3.63 U/L for control and F1, respectively. When these results were analyzed they were found in the safe limit. According to Sime, Quick, Saleh, and Martin (2007) the safe limit for monitoring of hepatocytic integrity was established ranging from 123.1 ± 7.67 to 183.8 ± 9.18 U/L in male *SD* rats and 72 ± 16 to 94 ± 32 U/L for female *SD* rats for ALP, whereas 88 ± 18.2 to 111 ± 50.6 U/L in males and 80 ± 9 to 89 ± 14 in females for AST, whereas for ALT normal range for male *SD* rats were ranged from 38 ± 3.9 to 48 ± 7.8 IU/L and for females ranged between 44.50 ± 7.37 to 53.70 ± 15.38. Current findings are similitude to the normal range of hepatic enzymes which is evident that no hepatocytic activity took place by the administration of the test formulations and results were also in concordance with the control fed experimental *SD* rats.

### 3.8 Sensory evaluation

The sensory evaluation of weaning mixes is very crucial because the consumer's acceptance is of prior importance in value addition and processing of food items. In the current study, weaning formulations were prepared from the date powder with the addition of different fortificants. Due to dark color of the date powder the color of dry mix was light brown in the first formulation due to higher contents of rice flour and whole milk powder. For color, texture, and ease of preparation, the control scored minimum score compared to the formulations. The highest position for color was fetched by F1 with value 12.61 ± 0.34 followed by F2 with value 11.64 ± 0.866. A similar trend was followed by the texture where value ranged from 11.72 ± 0.29 to 12.53 ± 0.33 for F1. For ease of preparation, the highest score was obtained by F2 with value 13.08 ± 0.38, the significant difference was exhibited by all the formulations. The value for smoothness and thickness was varied from 10.38 ± 0.23 to 11.56 ± 0.26, 10.49 ± 0.26 to 11.63 ± 0.28. The highest value of sweetness among the experimental formulations was in F3 with value 11.66 ± 0.23, it is evident from the sweetness contents that higher was the date powder contents higher will be the sweetness level. The taste and flavor or F2 formulations were observed as the best with values 12.61 ± 0.26 and 11.57 ± 0.28 among the formulations, the control has scored the best for flavor and on no second for taste with very little difference. The overall acceptability of the control was noted with value 12.72 ± 0.58 and among formulations; the highest acceptability was earned by the F2 formulation, that is, 12.55 ± 0.40.

**FIGURE 2:**
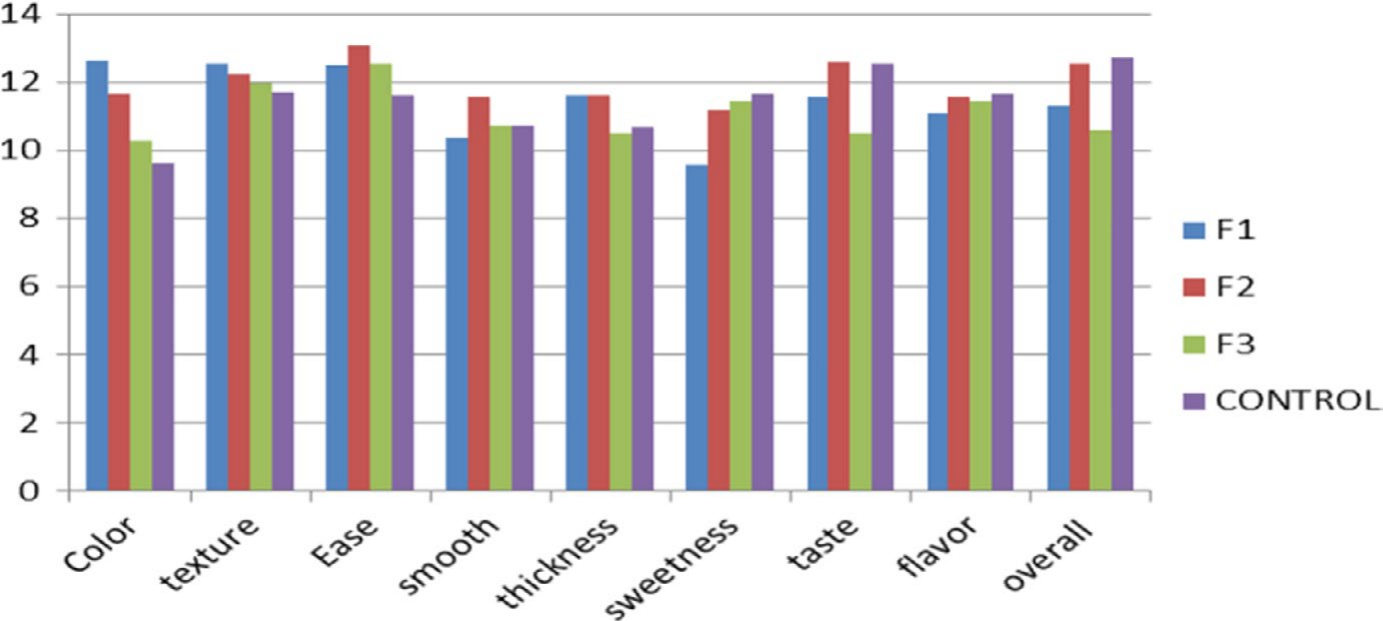
Mean value description for sensory evaluation of formulations and control

The lowest value for overall acceptability was noted in F3 formulation. It can be deduced that the higher thickness, darker color, and little bit bitter flavor can contribute to the overall acceptability of the F3 formulation due to higher contents of the date powder (Figure 2).

### 3.9 Conclusion

The amelioration of weaning food to uplift child health required novel approaches. The effort made in current study showed good results regarding weaning blends. Date powder contributed to protein contents, improve the micromineral and amino acid profile of the blends clearly, and the control with no date powder was lacking good protein quality, good amino acid score, and mineral contents. Therefore, date powder blending for weaning mixes preparation was proved in current study.
